# Mass Spectrometric Proteomics 3.0

**DOI:** 10.3390/ijms26136242

**Published:** 2025-06-28

**Authors:** Paolo Iadarola, Simona Viglio

**Affiliations:** 1 Department of Biology and Biotechnology “L. Spallanzani”, University of Pavia, 27100 Pavia, Italy; 2 Department of Molecular Medicine, University of Pavia, 27100 Pavia, Italy; simona.viglio@unipv.it

Mass spectrometry (MS) has undergone a profound transformation over the past two decades, expanding in both technical capability and scientific impact. Once primarily a tool for molecular weight determination, MS is now central to a wide range of disciplines, from proteomics and metabolomics to clinical diagnostics and environmental monitoring. This evolution has been driven by advances in high-resolution instrumentation, improved ionization techniques, and increasingly sophisticated data analysis platforms. As we enter what may be termed the “Mass Spectrometry 3.0” era, the field is being redefined by its integration with high-throughput workflows, artificial intelligence (AI), single-cell technologies, and real-time, in situ applications [1,2,3]. This Special Issue was conceived to capture and reflect on this pivotal phase. The articles collected here present the remarkable breadth and depth of modern MS-based research, particularly in proteomics, which has moved well beyond protein identification. Quantitative proteomics, single-cell analyses, post-translational modification (PTM) mapping, real-time metabolic flux tracking, and spatially resolved techniques expand the possibilities of what MS can achieve. More importantly, the integration of MS with other omics layers, such as transcriptomics, genomics, and metabolomics, facilitates integrating systems-level insights with complex biological processes [4]. At the same time, the field faces ongoing challenges. These include the need for enhanced sensitivity, greater specificity, robust data analysis pipelines, and standardized protocols, especially in clinical and regulatory settings [5]. The contributions in this Special Issue directly address these challenges. The featured studies present advances in sample preparation and enrichment, improved computational tools for data interpretation, and innovative strategies for accurate quantification in complex biological and clinical samples. Several articles also propose new metrics for evaluating proteome completeness and reproducibility, contributing to the development of best practices across the field. Looking ahead, the future of MS will be shaped by the convergence of analytical platforms, data science, and biology. Developments in miniaturization, real-time acquisition, and AI-driven data processing are expected to accelerate both the speed and accessibility of MS technologies. Furthermore, emerging directions such as ambient ionization, imaging MS, and lab-on-a-chip integration are paving the way toward real-time diagnostics and precision medicine at scale [6,7]. We hope this Special Issue serves not only as a snapshot of the current state of MS but also as a catalyst for new ideas and collaborations. The era of “Mass Spectrometry 3.0” is not defined by incremental progress alone; it is characterized by a reimagining of what is possible when this technology is embedded deep within molecular science.
